# Prospective evaluation of the VITOM 3D exoscope in ear surgery compared with surgical microscopes: part II—optical performance, handling, workload and ergonomics

**DOI:** 10.1007/s00405-026-10045-x

**Published:** 2026-03-09

**Authors:** Christoph Müller, Hai Yen Tao, Marcus Neudert, Nikoloz Lasurashvili, Thomas Beleites, Thomas Zahnert, Joseph Morgenstern

**Affiliations:** Department of Otorhinolaryngology, Head and Neck Surgery, Medical Faculty Carl Gustav Carus, University Hospital Dresden, Saxony, Germany

**Keywords:** Exoscope, Ear Surgery, Ergonomy, VITOM, Outcome parameters

## Abstract

**Background:**

The aim of this study was to evaluate the feasibility of a 3D exoscope system (VITOM 3D, Karl Storz, Tuttlingen, Germany) in comparison with conventional surgical microscopes (SM, hereafter referred to as ‘microscope’) in ear surgery. This paper represents the second part of a two-part publication series.

**Methods:**

A total of 62 patients were included in the study, with 31 cases assigned to the exoscope group (E+) and 31 to the conventional microscope group (E–). Surgical procedures comprised cochlear implantation (nE+ = 10, nE– = 10), reconstructive middle ear surgery for chronic otitis media with cholesteatoma (COMwC; nE+ = 11, nE– = 11), and without cholesteatoma (COMsC; nE+ = 10, nE– = 10). While the first part of the study focused on objective perioperative parameters, the present part specifically evaluates subjective outcomes as reported by the operating surgeons. These include technical aspects (perceived image quality assessed through a detailed questionnaire), surgical aspects (handling characteristics, mental and physical workload evaluated using the NASA-TLX questionnaire and ergonomic factors assessed using a questionnaire to assess localized musculoskeletal discomfort (LMD)), as well as overall satisfaction. All assessments were completed immediately after each procedure using standardized questionnaires. Additionally, a cluster analysis was performed to identify potential differences between the subgroups.

**Results:**

The microscope received significantly better scores regarding image quality. The exoscope achieved comparable ratings for body posture and ergonomic comfort, while handling was rated slightly inferior. NASA-TLX assessments revealed no significant differences in mental or physical workload between the systems. LMD scores indicated overall low musculoskeletal strain, with only the upper back showing slightly higher discomfort in the exoscope group. Cluster analysis demonstrated heterogeneous subgroup patterns without a consistent trend, although cochlear implantation procedures were most frequently associated with less favorable subjective ratings in the exoscope group. Direct comparison of both visualization systems confirmed a slight but significant advantage of the surgical microscope in terms of system operation, visual display, and handling, while ergonomics showed no significant difference

**Conclusions:**

Under the conditions evaluated in this study, the investigated exoscope system did not achieve equivalence to a standard microscope across all assessed domains. In particular, limitations in optical performance and handling currently preclude its use as a full substitute for the microscope in routine ear surgery. The present findings apply to the specific system generation evaluated and the study conditions employed. Ongoing technological advancements and further refinement of exoscope systems may enable future generations to achieve equivalence with microscopes and serve as viable alternatives in routine clinical practice

**Clinical trial registration:**

Our study involves a clinical trial registered at the ethics committee of the Carl Gustav Carus Faculty of Medicine at the Technische Universität Dresden, with the registration number EK 393102018. This registration reflects our commitment to transparency and adherence to international standards for clinical research.

**Supplementary Information:**

The online version contains supplementary material available at 10.1007/s00405-026-10045-x.

## Introduction

The microscope has been the gold standard in ear surgery for decades, but in recent years, exoscopes have been introduced as alternative visualization systems. Part I of this two-part prospective randomized study evaluated objective intraoperative parameters when using the VITOM 3D (Karl Storz, Tuttlingen, Germany) compared with conventional microscopes in cochlear implantation (CI) and reconstructive middle ear surgery in chronic otitis media with (COMwC) and without cholesteatoma (COMsC) [1]. The analysis demonstrated procedure-dependent differences and revealed that prolonged operating times associated with the exoscope were partly attributable to learning curves and technical handling, resulting in an overall inferiority of the exoscope.

However, objective measurements alone do not fully capture the complexity of surgical performance, as the subjective experience of the operating surgeon is also decisive for the acceptance and clinical applicability of a visualization system. The subjective experiences of the surgeon, such as perceived visualization quality, workload, and ergonomic strain, are equally important determinants for the integration of novel systems into routine clinical practice. Previous studies suggested that exoscopes may improve ergonomics [2–4] and enable shared visualization [2–6] but have also pointed to persistent limitations in depth perception, illumination, and overall image quality [2, 3, 7]. However, most of these investigations [2, 3, 6, 8] relied on anecdotal reports or limited assessments, and systematic evaluation of surgeons’ perspectives remains scarce.

Therefore, the present paper, Part II of this study, focuses on subjective outcome parameters. In contrast to Part I, which analyzed quantitative time-based data, this study evaluates surgeons’ perceptions of visualization quality, ergonomics, and handling during CI and reconstructive middle ear surgery of COMwC and COMsC. By addressing these subjective aspects, the analysis aims to complement the objective findings and provide a more comprehensive assessment of the advantages and limitations of the VITOM 3D compared with the conventional microscope in otologic surgery. It should be emphasized that the conclusions presented here are based exclusively on subjective surgeon-reported outcomes. Findings from Part I are referenced solely to provide contextual background, particularly with respect to operating time and learning-curve effects, and do not constitute primary endpoints of the present analysis.

## Materials and methods

For details on the study design, patient cohorts and applied statistical methods please refer to the published results and the appendices of the first part of this study [1].

### Study design

This paper represents the second part of a prospective randomized trial comparing the VITOM 3D (Karl Storz, Tuttlingen, Germany) with conventional microscopes in otologic surgery. The study was conducted at a tertiary referral hospital between October 2019 and September 2020.

The trial consisted of two arms: the exoscope arm (E+) and the microscope arm (E–). Each arm included three surgical subgroups with different levels of standardization: cochlear implantation (CI), reconstructive middle ear surgery for chronic otitis media with cholesteatoma (COMwC), and without cholesteatoma (COMsC). In total, 62 patients were enrolled (nE + = 31; nE– = 31), comprising 10 CI, 11 COMwC, and 10 COMsC procedures per study arm.

The sample size was calculated on the basis of the primary endpoint of the overall project (cut–suture time, see Part I). Assuming a clinically acceptable increase of 20.0% in cut–suture time, a type I error rate of α = 0.025, and a power of β = 0.9, the required sample size was estimated as *n* = 27 per arm. To account for potential dropouts, 62 patients were finally included. Please refer to the published results of the first part of this study for a detailed analysis of the primary endpoint.

Seven surgeons participated in the study (one resident and six senior physicians). Two senior surgeons (NL and TB) performed the majority of the procedures (48 out of 62; 77.4%), ensuring a consistent level of expertise, while residents were involved under supervision.

The study followed the tenets of the Declaration of Helsinki and was approved by the Institutional Review Board (IRB00001473) at the Technische Universität Dresden (EK 393102018).

### Subjective parameters

In addition to the objective parameters reported in Part I of this publication series, subjective perioperative parameters were assessed in order to evaluate visualization quality, handling, workload, and ergonomics from the perspective of the operating surgeons. For this purpose, structured self-developed questionnaires were administered immediately after each procedure (see Appendix A1 to A4). In addition, all surgeons hat to complete the NASA Task Load Index (NASA-TLX, see Appendix A5) [9] and a questionnaire on localized musculoskeletal discomfort (LMD, see Appendix 6) according to Kuorinka and Andersson, modified after Borg and Hamberg-van Reenen [10–12] upon completion of surgery.

### Questionnaires

*Self-developed questionnaire part 1 and 2*.

A self-developed questionnaire (Appendix A1 to A3) was designed to address general and procedure-specific aspects of surgical visualization. The first part of the questionnaire covered general questions about domains such as depth perception, visibility of details, image representation, illumination, handling of the system, body posture during the procedure whereas the second part was procedure-specific (CI, COMwC, or COMsC) and focused on anatomical landmarks of particular relevance to the surgery performed. During the course of the study, the questionnaire was supplemented by an additional parameter regarding disturbing optical artifacts due to the absence of an ocular effect when operating via monitor view (question 10).

Responses for the first and second part were recorded on a six-point Likert scale (1 = very good/very satisfied to 6 = very poor/very dissatisfied, first and second part of the questionnaire). In addition (third part), four visual analogue scales (VAS) were used for direct comparisons between the microscope and the exoscope regarding handling, visual representation, ergonomics, and usability.

*Self-developed questionnaire part 3: Overall assessment of both systems*.

Following these general and surgery-specific questions, four additional items (see Appendix A4) addressed the direct comparison of the two visualization systems, ‘microscope’ and ‘VITOM 3D’. Respondents were asked to indicate which system they considered superior with regard to overall “operation”, “ergonomics”, “visual representation” and “handling”. A rating scale divided into ten equal segments was used. The microscope was positioned on the left end of the scale and the VITOM 3D on the right. The further to the left or right the mark was placed, the stronger the preference for the respective system; the midpoint represented equal evaluation.

*NASA Task Load Index (NASA-TLX)*.

To capture subjective workload, the NASA Task Load Index (NASA-TLX) was applied (see Appendix A5). This validated and widely used instrument was originally developed for aviation but has since been established in healthcare settings. It evaluates six dimensions: mental demand, physical demand, temporal demand, perceived performance, effort, and frustration. Each dimension is scored on a 20-point visual analogue scale anchored by bipolar descriptors.

For the present study, the unweighted raw version (RTLX) was used, which omits the weighting procedure of the original version. This modification has been shown to maintain sensitivity while improving feasibility and time efficiency in clinical studies [9].

*Localized Musculoskeletal Discomfort (LMD)*.

Musculoskeletal strain was assessed using the Localized Musculoskeletal Discomfort (LMD) questionnaire (see Appendix A6), adapted from Hamberg-van Reenen [12] based on the original investigations by Kuorinka and Andersson [11] and the Category Ratio (CR-10) scale published by Borg [10]. Surgeons rated the intensity of discomfort for predefined body regions relevant to the seated surgical position (neck, shoulders, upper back, lower back, and arms). The scale ranged from 0 (“no discomfort”) to 10 (“extremely strong discomfort, nearly maximal”). A score of 5 corresponds to half a score of 10. Except for the rating of 0.5 (extremely little discomfort), only round numbers were presented. To ensure consistent reporting, a body chart was provided, and ratings were assigned to the corresponding regions.

### Statistical analysis

All analyses were performed using IBM SPSS^®^ Statistics 27 (IBM, Armonk, USA). Results are presented as mean ± standard deviation (SD). A significance level of *p* ≤ 0.05 was applied, with * p < 0.05 (significant), ** p < 0.01 (highly significant), and *** p < 0.001 (highly significant) indicating levels of significance. Normal distribution was tested with the Kolmogorov–Smirnov and Shapiro–Wilk tests. Since normality could not be confirmed in several cases, group comparisons were performed using the non-parametric Mann–Whitney–U test. Overall results refer to comparisons between the exoscope (E+) and microscope (E–), whereas subgroup analyses were conducted separately for CI, COMwC, and COMsC.

Throughout the manuscript, the term ‘significant’ refers to statistical differences at a significance level of *p* < 0.05. Non-significant differences are described as ‘tendencies’ or ‘trends.’

*Statistical Cluster Analysis*.

To assess similarity patterns between the three surgical subgroups (CI, COMwC, COMsC) across the two visualization systems, a hierarchical cluster analysis was performed using Ward’s minimum variance method based on Euclidean distances between subgroup mean vectors. For each domain (General questionnaire parameters, NASA-TLX and Localized Musculoskeletal Discomfort (LMD)), the incremental increase in within-cluster variance after merging two clusters was computed as

$$\:\varDelta\:SSE\left(A,B\right)=\frac{{n}_{A}{n}_{B}}{{n}_{A}+{n}_{B}}{||{\mu\:}_{A}{-\mu\:}_{B}||}^{2}$$.

resulting in two linkage distances (ΔSSE₁ and ΔSSE₂). Their ratio $\:R=\:\frac{{\varDelta\:SSE}_{2}}{{\varDelta\:SSE}_{1}}$ quantified the distinctness of the third subgroup. Smaller ΔSSE values indicate similar subgroups, whereas larger values reflect increasing dissimilarity.

Accordingly, ratios *R* ≈ 1.0 denote comparable similarity among all subgroups, *R* > 1.5 suggest moderate differentiation, and *R* > 2.0 indicate a clearly distinct subgroup. Cluster metrics were visualized using bar and scatter plots. All analyses were implemented in Python (v3.10) using NumPy, SciPy, pandas, matplotlib, and seaborn. Please see Appendix 7 for details regarding the methodological approach of the applied cluster analysis, calculated values and the applied Python script.

## Results

### General section of surgeons’ questionnaire (Part 1)

**Fig. 1:**
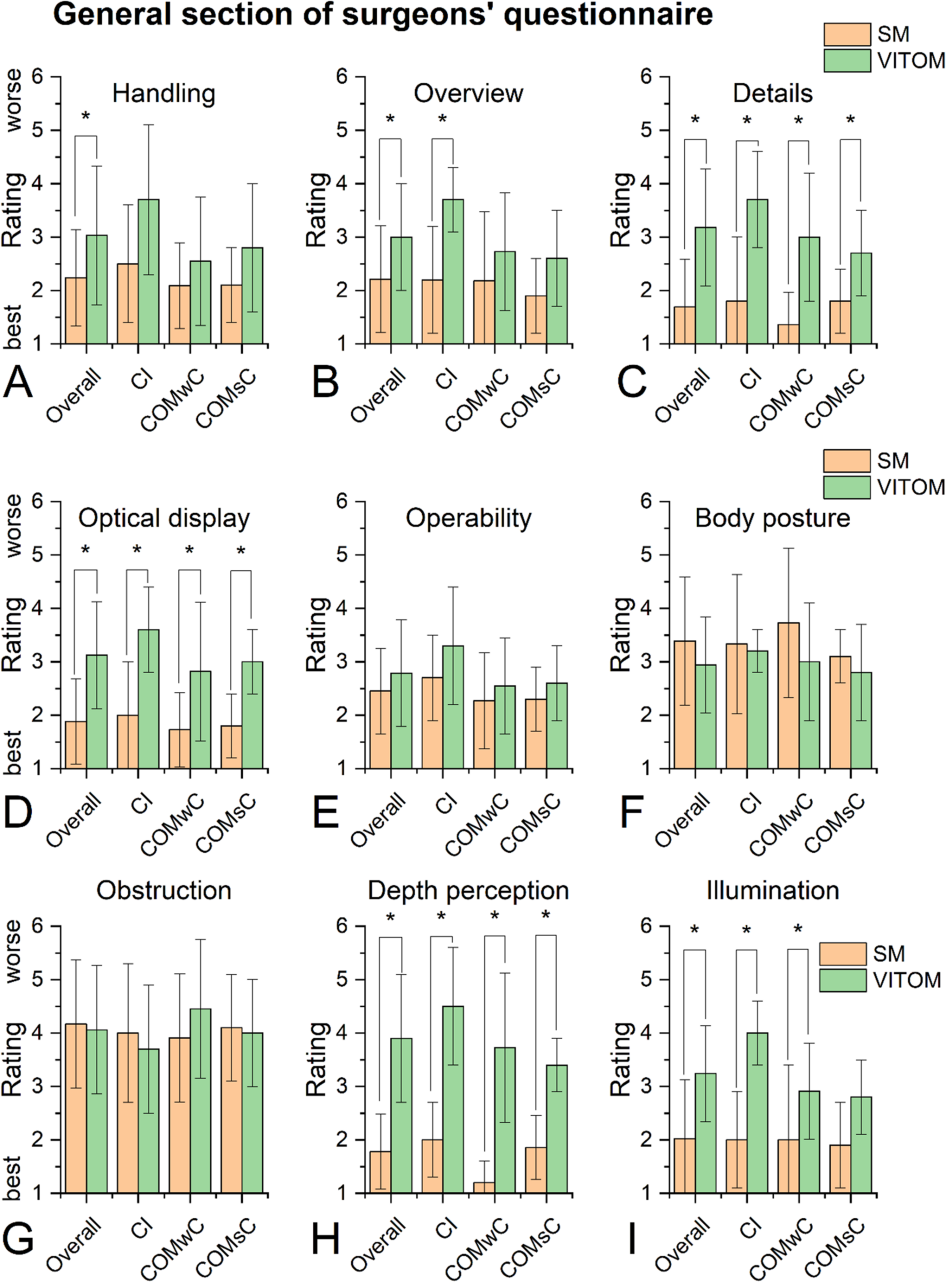
General section of the surgeons’ questionnaire comparing the exoscope (VITOM 3D) and the microscope across the overall cohort and the surgical subgroups cochlear implantation (CI), chronic otitis media with cholesteatoma (COMwC), and without cholesteatoma (COMsC). Items assess general aspects of visualization quality, system handling, and surgeon posture. Ratings were recorded on a six-point Likert scale (1 = very good/very satisfied; 6 = very poor/very dissatisfied), with lower values indicating more favorable ratings. Except for ‘body posture’ (question 6) and ‘obstruction’ (question 7; CI and COMsC), the exoscope showed tendentially lower or significantly lower ratings than the microscope across the overall cohort and all subgroups. Bars represent mean values with standard deviations. Statistically significant differences between systems (*p* < 0.05) are marked with an asterisk (*)

Due to the extensive scope of the questionnaire analysis, only the most relevant items—those addressing system handling, optical performance, and the surgeon’s body posture during surgery—are presented here. As summarized in Fig. 1, subjective ratings revealed clear differences between the two visualization systems. With the exception of the items ‘body posture’ (question 6; entire cohort and subgroups) and ‘obstruction’ (question 7; entire cohort and the subgroups CI and COMsC), the exoscope tended to perform worse or was significantly inferior to the microscope (SM) across all analyses. This applied to both the overall study cohort (mean value across the subgroups CI, COMwC, and COMsC) and the individual subgroups themselves.

Significant inferiority of the exoscope was particularly observed in terms of optical properties. This included the overview of the surgical field (question 2, entire cohort and subgroup ‘CI’), visibility of details (question 3, entire cohort and all subgroups), optical display (question 4, entire cohort and all subgroups), depth perception (question 8, entire cohort and all subgroups) and illumination (question 11, entire cohort and subgroups ‘CI’ and ‘COMwC’). For the item ‘handling’ (question 1), the exoscope also showed a significant inferiority compared with the SM in the entire cohort.

### Specific section of surgeons’ questionnaire (Part 2)

**Fig. 2:**
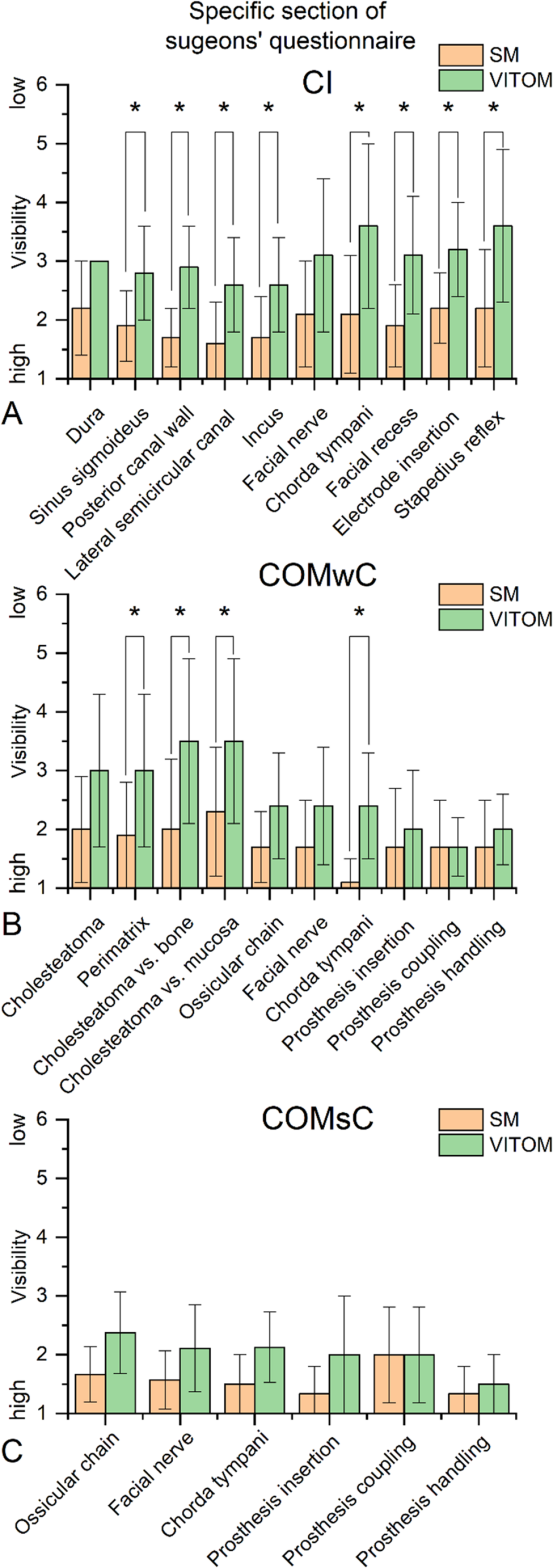
Specific section of the surgeons’ questionnaire comparing the exoscope (VITOM 3D) and the microscope for all three surgical subgroups: cochlear implantation (CI), chronic otitis media with cholesteatoma (COMwC), and without cholesteatoma (COMsC). Items assess the visibility and handling of procedure-specific anatomical landmarks. Ratings were recorded on a six-point Likert scale (1 = very good; 6 = very poor), with lower values indicating more favorable ratings. Except for ‘prosthesis coupling’ in the COMwC and COMsC subgroups, the exoscope received tendentially lower or significantly lower ratings than the microscope. Bars represent mean values with standard deviations. Statistically significant differences between systems (*p* < 0.05) are marked with an asterisk (*)

The procedure-specific questionnaires (Fig. 2**)** revealed differences between the two visualization systems depending on the surgical indication.

In cochlear implantation (CI), almost all assessed landmarks were rated significantly better when using the microscope. This applied to the visualization of the sigmoid sinus, posterior canal wall, lateral semicircular canal, incus, chorda tympani, facial recess, electrode insertion, and the stapedius reflex. Only for the visibility of the dura and the facial nerve was a tendency towards superiority of the microscope observed.

In cholesteatoma surgery (COMwC), the microscope showed significant superiority in the assessment of the perimatrix, the differentiation of cholesteatoma from bone and mucosa, and the visibility of the chorda tympani. All other items, except for ‘prosthesis coupling’ (which showed equal ratings between the VITOM 3D and the microscope), tended to favor the microscope.

For reconstructive surgery without cholesteatoma (COMsC), all items, except for ‘prosthesis coupling’ (which showed equal ratings between the VITOM 3D and the microscope), tended to favor the microscope.

### NASA-TLX

**Fig. 3:**
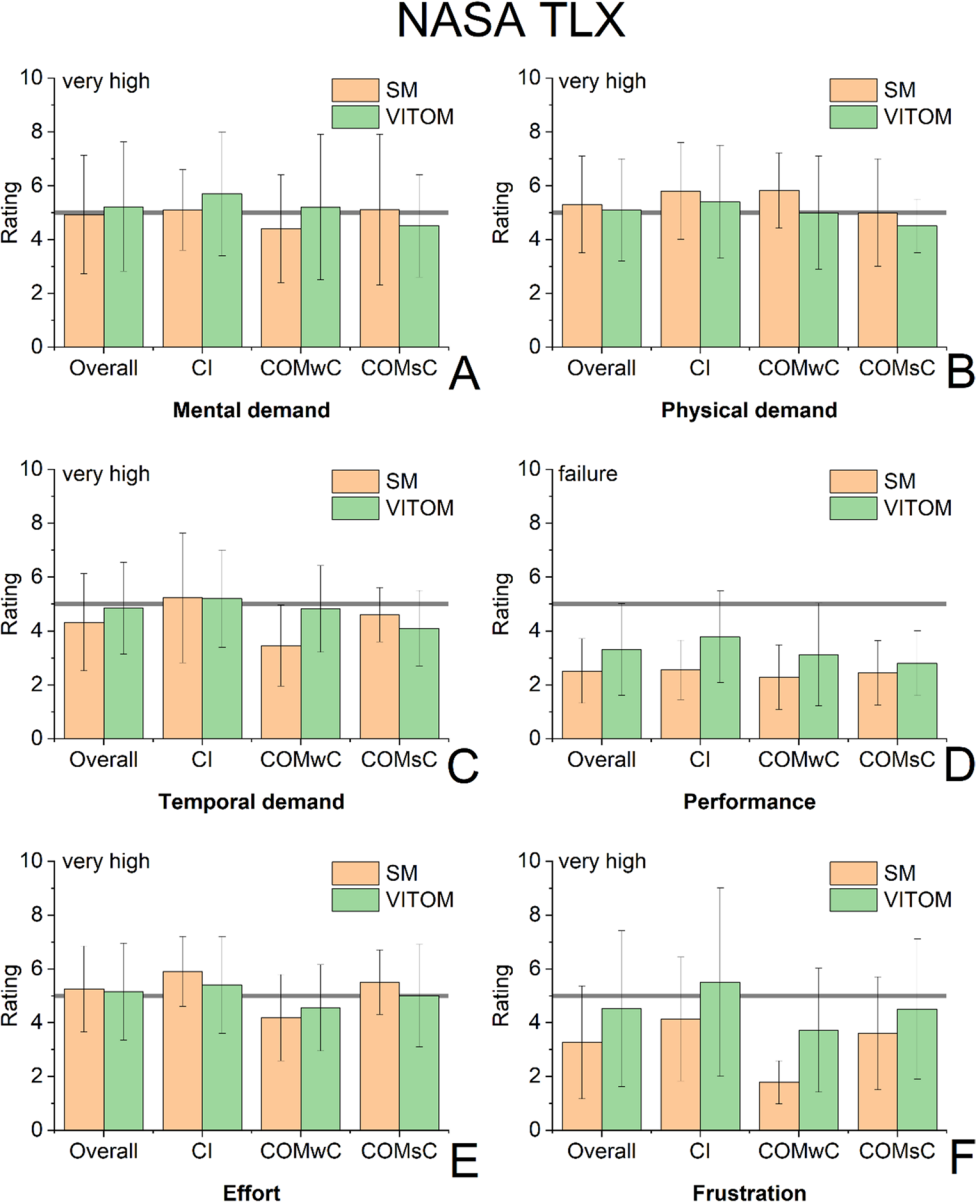
NASA Task Load Index (NASA-TLX) assessment comparing the microscope and the exoscope (VITOM 3D) for the overall cohort and the surgical subgroups cochlear implantation (CI), chronic otitis media with cholesteatoma (COMwC), and without cholesteatoma (COMsC). The unweighted raw NASA-TLX (RTLX) was used to assess mental demand, physical demand, temporal demand, perceived performance, effort, and frustration. Scores were recorded on a 10-point visual analogue scale for each dimension. No statistically significant differences were observed between the two visualization systems across any dimension or subgroup. Bars represent mean values with standard deviations

As illustrated in Fig. 3, no significant overall differences were found between the two visualization systems. For the entire cohort, ratings for the VITOM 3D were generally comparable to those for the microscope in terms of ‘effort’, slightly lower for ‘physical demand’, and slightly higher for the remaining four dimensions (‘mental demand’, ‘temporal demand’, performance’, ‘frustration’). In the subgroup analysis, higher ratings for the microscope were observed only for ‘mental demand’ (COMsC subgroup), for ‘physical demand’ (across all subgroups), for ‘temporal demand’ (CI subgroup), and for ‘effort’ (CI and COMsC subgroups), whereas no subgroup differences were seen for ‘performance’ and ‘frustration’. In all other subgroups and dimensions, higher ratings were observed for the VITOM 3D.

### LMD

**Fig. 4:**
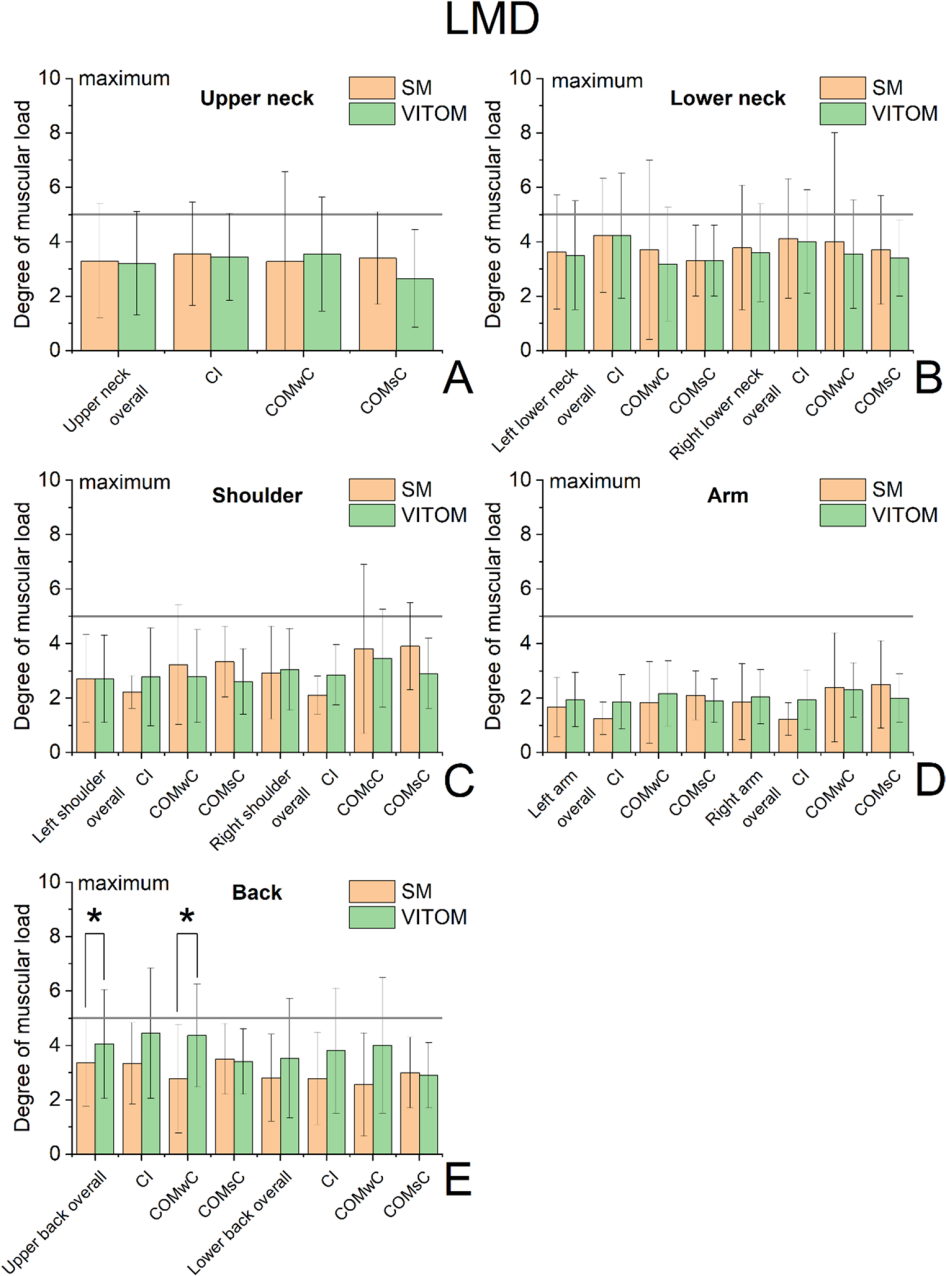
Localized musculoskeletal discomfort (LMD) assessment comparing the microscope and the exoscope (VITOM 3D) for the overall cohort and the surgical subgroups cochlear implantation (CI), chronic otitis media with cholesteatoma (COMwC), and without cholesteatoma (COMsC). Discomfort was rated for the following body regions: upper neck, left and right lower neck, left and right shoulder, left and right arm, and lower back. Ratings range from 0 (no discomfort) to 10 (extremely strong discomfort). A reference line indicates a score of 5, corresponding to strong discomfort and half the intensity of the maximum score (10). Statistically significant differences between systems (*p* < 0.05) were observed for the upper back in the overall cohort and the COMwC subgroup and are marked with an asterisk (*). Bars represent mean values with standard deviations

As illustrated in Fig. 4, significant statistical differences were observed only for the upper back (entire cohort and COMwC subgroup), with slightly higher strain in the VITOM 3D study arm. For the remaining parameters, ratings for the VITOM 3D were generally comparable to those for the microscope in terms of the upper neck, left lower neck, and both shoulders, slightly lower for the right lower neck, and slightly higher for the left and right arm and the lower back.

No mean value in the entire cohort or in any subgroup exceeded a score of 5, which corresponds to ‘high discomfort’.

Subgroup analyses showed procedure-specific patterns. In COMsC, the exoscope received slightly better ratings in most regions, with only minimal differences in the neck/shoulder and lower back. In CI surgery, the exoscope was rated better in the neck and shoulders, but the back regions were rated more favorably with the microscope. The largest differences were seen in COMwC, where the microscope was rated significantly better for the upper back and nearly significant for the lower back, while shoulder regions favored the exoscope or showed no difference.

### Cluster analysis and overall assessment of both systems

**Fig. 5:**
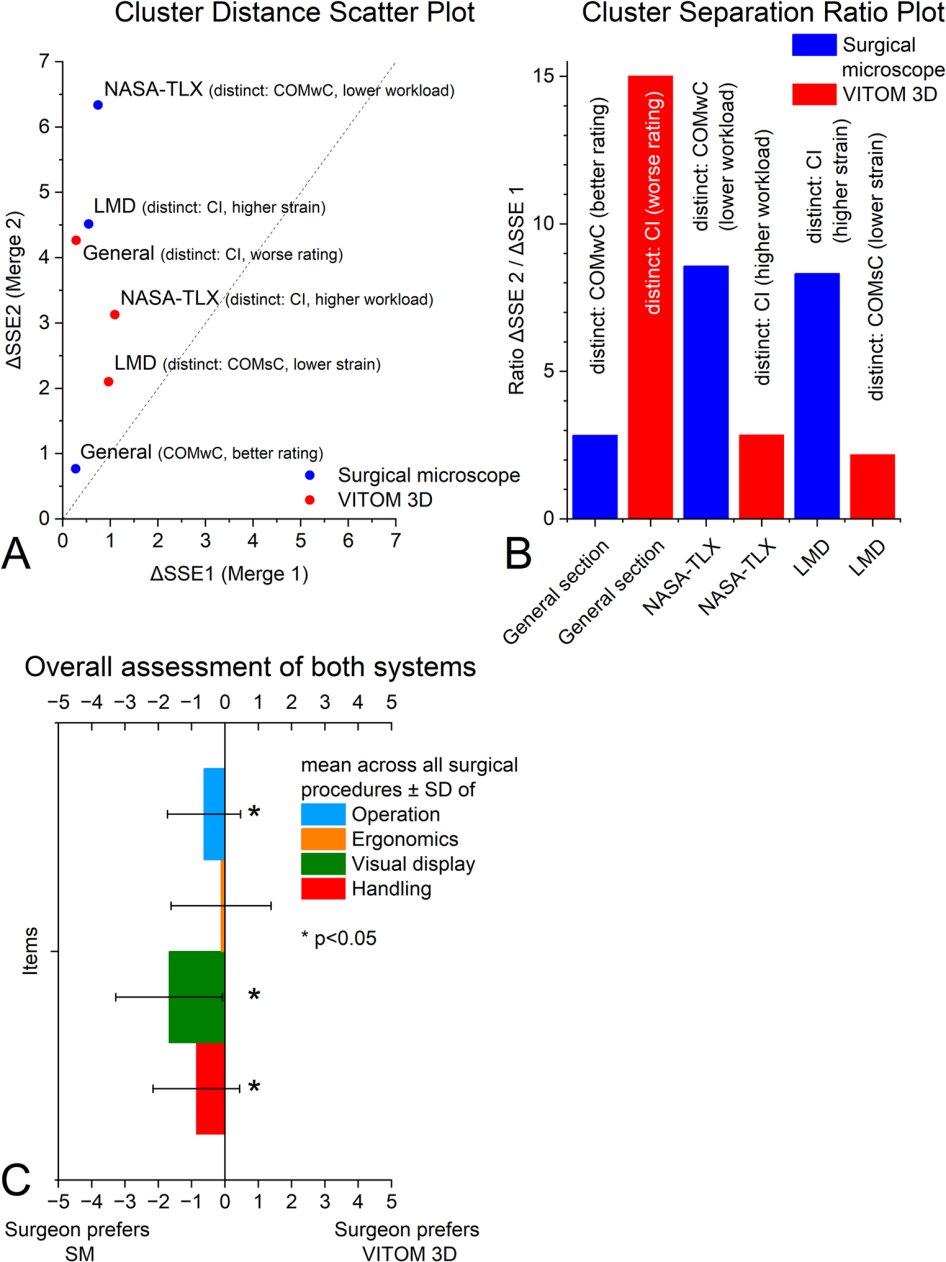
Exploratory cluster analysis and direct comparison of visualization systems. (**A**) Cluster distance scatter plot illustrating subgroup similarity based on integrated subjective outcomes across different measurement domains (general questionnaire, NASA-TLX, and localized musculoskeletal discomfort). (**B**) Cluster separation ratio plot (R = ΔSSE₂/ΔSSE₁), with higher values indicating greater distinctiveness of a surgical subgroup relative to the remaining clusters. In three out of six cluster analyses, the cochlear implantation (CI) subgroup emerged as the most distinct cluster and was associated with less favorable subjective ratings. (**C**) Direct comparison of the exoscope (VITOM 3D) and the microscope with respect to system operation, ergonomics, visual display, and handling, assessed using a visual analogue scale. Bars represent mean values with standard deviations. Statistically significant differences between systems (*p* < 0.05) are marked with an asterisk (*)

Hierarchical cluster analysis revealed distinct subgroup patterns depending on both the domain and visualization system. The cluster distance scatter plot (Fig. 5A) and the cluster separation ratio plot (Fig. 5B) illustrated that the magnitude of the second linkage distance (ΔSSE₂) and the corresponding cluster separation ratio (R = ΔSSE₂ / ΔSSE₁) varied across domains (general questionnaire, NASA-TLX, LMD). Overall, the microscope condition showed higher separation ratios (*R* ≈ 8–9) for NASA-TLX and LMD, indicating stronger differentiation between the clusters, while the VITOM 3D system produced more homogeneous clusters (*R* ≈ 2–3). Regarding the general questionnaire, *R* ≈ 15 in the microscope study arm suggested a pronounced outlier behavior of the most distinct subgroup/cluster (*R* ≈ 3 in the exoscope study arm). Regarding the analysis of the most distinct subgroups (CI, COMwC, COMsC) across both visualization systems and measurement domains (General questionnaire, NASA-TLX, and LMD), a highly heterogeneous pattern without a consistent trend was observed. Notably, in three out of six cluster analyses, the CI subgroup emerged as the most distinct cluster, consistently associated with less favorable subjective ratings—showing markedly poorer ratings in the general questionnaire (exoscope study arm, *R* ≈ 15), slightly higher perceived workload in the NASA-TLX (exoscope study arm, *R* ≈ 4), and substantially higher musculoskeletal strain in the LMD (microscope arm, *R* ≈ 9). In two out of six cluster analyses, the COMwC subgroup emerged as the most distinct cluster, being associated with more favorable subjective ratings—showing slightly better scores in the general questionnaire (microscope study arm, *R* ≈ 3) and markedly lower perceived workload in the NASA-TLX (microscope study arm, *R* ≈ 9). Please refer to Table A1 in Appendix A7 for a detailed overview of the calculated cluster metrics and corresponding values.

The direct comparison of the two visualization systems included four parameters: ‘system operation’, ‘ergonomics’, ‘visual display’, and ‘overall handling’, with ratings ranging from − 5 (microscope better) to 0 (equal) to + 5 (exoscope better) (Fig. 5C). ‘System operation’ was rated slightly but significantly in favor of the microscope. Nearly half of all respondents selected the neutral category, while some indicated a preference for the microscope and a smaller proportion favored the exoscope. ‘Ergonomics’ showed no significant difference between the two systems. More than half of the participants rated both systems equally, and the remaining responses were distributed almost evenly between the microscope and the exoscope. For ‘visual display’, the mean rating shifted towards the microscope, with a significant difference between the two systems. While both systems covered the full range of possible ratings, the most frequent response was a slight preference for the microscope, followed by stronger ratings on the same side of the scale. For ‘handling’, the mean rating was again slightly but significantly in favor of the microscope. The neutral category was chosen most frequently—by nearly half of all respondents—while the second most common response favored the microscope. Only a few participants indicated a preference for the exoscope.

## Discussion

Since 2019, an increasing body of evidence has documented the clinical adoption of exoscopes, positioning them between the microscope and the endoscope, with neurosurgery taking a pioneering role in their clinical application [13–15] and in surgical training [16].

The routine use of exoscopes in the field of otorhinolaryngology, head and neck surgery appears to lag behind that in neurosurgery when considering the volume of published studies [17]. Nevertheless, data are also available in this discipline documenting their application compared to the microscope across major subspecialties, including lateral skull base and ear surgery [1, 4, 5, 18–27], salivary gland surgery [26, 28], transoral procedures involving the oral cavity, pharynx, and larynx, as well as neck surgery including plastic and reconstructive surgery [26, 29–34].

A review of available studies has identified three major domains that need to be assessed when comparing the performance of microscopes and exoscopes. First, *technical aspects* include optical properties (e.g., image quality, magnification, illumination, stereopsis, depth of field) as well as system-related factors such as connectivity, acquisition cost, and running expenses. Second, *surgical aspects* cover handling characteristics, available functionalities that enhance intraoperative performance (e.g., robotic assistance, image enhancement, AI-based tools), as well as ergonomic factors and mental and physical workload. Third, *educational aspects* refer to the system’s potential as a teaching and training tool.

In our study, the first and second domains were primarily addressed through questionnaire-based evaluation. The following section therefore compares our findings with previously published data on the comparative use of exoscopes and microscopes in lateral skull base and ear surgery.

### Technical aspects

*Inferior optical performance of the VITOM 3D compared to the microscope*.

The largest differences between the two visualization systems concerned depth perception, detail discrimination, optical representation, and field illumination. These domains are fundamental to surgical visualization and were consistently rated worse for the exoscope.

Surgeons reported difficulties in assessing drilling depth, distinguishing between delicate structures, and maintaining stable lighting, with the image frequently perceived as either too dark or overexposed. One likely explanation lies in the limited dynamic range and sensitivity of the camera sensor, which is less adaptive than the optical path of a microscope. This challenge has also been highlighted in prior work, where insufficient brightness control and poor depth cues were reported as recurring limitations of exoscopic systems [3–5] .

A further factor may be the indirect viewing concept itself: while the microscope provides true stereoscopic vision via optics, the exoscope depends on digital stereoscopy and monitor-based perception. Subtle differences in resolution, latency, or depth cue rendering may therefore translate into reduced three-dimensional orientation during microsurgical steps. The wider variance of ratings in our cohort also suggests that surgeons’ individual adaptability to heads-up 3D vision plays a role.

*Procedure-specific questions regarding visibility of structures*.

CI surgery produced the highest number of significant differences. Except for dura and facial nerve visualization, most other landmarks—including major bony landmarks, ossicular structures, the facial recess, and electrode insertion—were rated inferior with the exoscope. The high degree of standardization of CI procedures likely amplifies these differences: surgeons are accustomed to a precise sequence of visual and manual steps, making deviations in optical quality particularly noticeable. Furthermore, the narrow surgical corridor in CI surgery places high demands on illumination and depth perception, two areas where the exoscope performed weakest.

In COMwC surgery, the differentiation of cholesteatoma from bone and mucosa proved especially challenging with the exoscope. This aligns with previous findings that exoscopes struggle to distinguish between tissues of similar brightness due to limited contrast and resolution [4]. Unlike the microscope, which provides a continuous optical image with high dynamic range, the exoscope depends on digital sensors and image processing, which may oversaturate bright surfaces (e.g., bone) while flattening subtle textural differences. As successful cholesteatoma removal relies on the precise delineation of disease margins, particularly at the interface with bone and mucosa, these limitations represent a significant drawback. Fine structures such as the perimatrix or the chorda tympani are easily obscured under suboptimal illumination or digital overexposure, contributing to lower ratings.

In COMsC, no significant differences emerged between systems. This is plausibly explained by the absence of cholesteatoma, which reduces the demand for fine tissue differentiation. The reconstructive steps in COMsC rely more on anatomical orientation and prosthesis handling than on subtle tissue contrast, making them less sensitive to the optical limitations of the exoscope.

*Effects of digital image processing*.

As a digital system, the exoscope inherently relies on sensor and processor performance. Surgeons in our study frequently criticized unnatural color reproduction, discontinuous zooming, and pixelation at higher magnification levels. These shortcomings likely stem from the absence of optical zoom and the dependence on digital scaling, which reduces resolution as magnification increases. Comparable issues have been reported by other groups, including unnatural color rendering and latency effects [2, 4].

Even a small image delay can disrupt fine motor control and may lead to visual discomfort or nausea in susceptible individuals. Moreover, the constant need to readjust lighting and focus to compensate for overexposure or reduced contrast appears to contribute to the lower overall scores of the exoscope. Together, these findings underscore that image processing technology remains the primary limiting factor of the VITOM 3D.

### Surgical aspects

*Mental and physical load (NASA-TLX)*.

NASA-TLX scores revealed no statistically significant differences between the systems, indicating comparable subjective mental and physical workload under the conditions studied. Although minor non-significant trends toward higher mental and temporal demand as well as frustration and performance were observed in individual dimensions and subgroups, these should not be overinterpreted. Such tendencies may reflect the need to adapt to changing image quality, repeated scope adjustments, and interpretation of digitally mediated visual information, particularly during early use of a novel visualization system. Ajmera et al. [18] reported lower NASA-TLX–based workload (reduced fatigue/effort, physical strain, and weakness) with the RoboticScope versus the operating microscope in otologic surgery. In a cadaveric otologic training model, Testa et al. [25] reported NASA-TLX ratings that favored the microscope over the VITOM 3D, with higher physical and temporal demand, effort, and frustration under the exoscope. Overall, the absence of statistically significant differences suggests that exoscope use did not substantially increase perceived cognitive or physical load, while observed tendencies are likely influenced by inter-individual variability and early user experience and should be interpreted with caution.

*Ergonomic aspects (LMD)*.

With regard to ergonomics, localized musculoskeletal discomfort (LMD) scores were overall low across both visualization systems and all surgical subgroups, with no mean value exceeding a score of 5, corresponding to strong discomfort. This finding indicates generally low subjective musculoskeletal strain under the conditions studied.

With the exoscope, a statistically significant increase in strain was observed in the upper back (overall assessment and subgroup analysis of COMwC). However, the absolute differences were small and should be interpreted with caution. This pattern may plausibly be related to the longer operating times reported for the exoscope in Part I of this study [1], which may have counteracted potential ergonomic benefits by increasing static postural load over time.

Apart from this finding, posture-related ratings and musculoskeletal discomfort indicated a general trend toward equivalence between the VITOM 3D and the microscope. Although heads-up visualization promotes a more upright posture and may reduce musculoskeletal strain, this advantage—reflecting the ergonomic principle of maintaining a neutral spinal alignment with reduced cervical and shoulder load—may only become clinically relevant once operating times approach those of the microscope. Previous studies have demonstrated improved ergonomics and decreased fatigue with exoscope use [7, 35]; however, these benefits were less pronounced in our cohort, likely due to longer procedure duration and the need for frequent repositioning and focus adjustments [1], which may have offset potential ergonomic gains.

While the LMD questionnaire reflects the individual’s subjective perception of ergonomic strain, objective tools such as the Rapid Upper Limb Assessment **(RULA)** provide a standardized assessment of biomechanical load. Accordingly, several studies have reported significantly lower RULA scores for exoscopic or hybrid visualization systems, indicating improved posture and ergonomics. Crimi et al. [20] and Lin et al. [23] found significantly lower RULA scores when using the Modus V and ORBEYE exoscopes, respectively, in ear and lateral skull base surgery. Lingl et al. [26] and Ajmera et al. [18] likewise demonstrated superior ergonomic performance with the KINEVO 900 (exoscope or hybrid approach) and RoboticScope, respectively, during ear and head and neck surgery.

### Further study limitations

An important limitation of the present study is that the primary endpoints in this second part of the two-part publication series are subjective and surgeon-reported. Even within a randomized design, such assessments are susceptible to bias. In particular, expectation effects and familiarity bias may have influenced ratings, as participating surgeons had long-standing routine with conventional microscopes. In addition, individual adaptability to heads-up 3D visualization may vary substantially and affect perceived image quality, handling, and confidence during surgery, potentially contributing to the observed heterogeneity across domains and surgical subgroups. Consequently, subjective ratings should be interpreted in the context of these potential sources of bias.

Learning-curve effects are also likely to have influenced subjective outcomes. Although learning effects were addressed in Part I using objective perioperative parameters, they remain equally relevant for surgeon-reported perceptions of handling, image quality, and overall confidence when using a novel visualization system. Early experience with an exoscope may be associated with increased cognitive effort, reduced subjective confidence, and more critical appraisal of visual performance. Accordingly, the present subjective ratings should be interpreted as reflecting an early adoption phase, with the expectation that perceptions may evolve with increasing exposure, dedicated training, and growing routine in heads-up 3D visualization.

Another limitation is that all procedures were performed at a single tertiary referral center, with a substantial proportion conducted by two senior surgeons. While this ensured a high level of procedural consistency, the findings may not be directly generalizable to lower-volume centers, training environments, or surgeons with different levels of experience. Subjective perceptions of visualization quality, handling, and workload may therefore differ in less specialized settings, and future multicenter studies including a broader range of institutions and experience levels are warranted.

Finally, a methodological limitation is that a substantial part of the subjective assessment relied on self-developed questionnaires that have not undergone formal validation. This approach was chosen because existing validated instruments do not capture key aspects of otologic visualization quality, such as depth perception, illumination of narrow surgical corridors, visibility of fine middle ear structures, or procedure-specific anatomical landmarks. To mitigate this limitation, validated and widely established instruments were deliberately included, namely the NASA Task Load Index (NASA-TLX) for subjective workload and the Localized Musculoskeletal Discomfort (LMD) questionnaire for ergonomic assessment. Nevertheless, the use of non-validated questionnaires should be considered when interpreting the subjective results.

### Cluster analysis and direct comparison of visualization systems

The cluster analysis was performed as an exploratory tool to identify patterns of similarity and heterogeneity across surgical subgroups beyond conventional subgroup-wise comparisons. While standard statistical analyses assess differences in individual outcome measures, the cluster approach integrates multiple subjective domains simultaneously (general questionnaire parameters, workload, and musculoskeletal discomfort), thereby providing a holistic view of subgroup behavior. This exploratory analysis suggests heterogeneous, procedure-dependent subjective perception patterns, most notably in cochlear implantation, and is intended to generate hypotheses rather than definitive subgroup effects.

When directly comparing both visualization systems, surgeons favored the microscope in terms of handling, optical display, and system operation. Interestingly, ergonomics were rated nearly equivalent, suggesting that the heads-up concept of the exoscope may indeed compensate for its optical shortcomings by improving posture. These mixed results indicate that, while the overall usability of the exoscope is acceptable, its visual performance remains the key limiting factor for broader clinical adoption.

### Outlook

Beyond the VITOM 3D evaluated in this study and as already mentioned above, several advanced exoscope systems are now available on the market, including the current VITOM generation (VITOM Eagle, Karl Storz) [34], AEOS (Aesculap) [36], ORBEYE (Olympus) [19], Modus V (Synaptive Medical) [20], RoboticScope (BHS Robotics) [18]. In addition, hybrid visualization platforms such as the KINEVO 900 (Carl Zeiss) combine the features of a conventional microscope with digital exoscopic functionality.

In addition to further improvements in image quality, current developments increasingly focus on robotic integration, image enhancement techniques, AI-based assistance tools, as well as connectivity and interoperability between systems.

## Conclusion

The VITOM 3D exoscope represents a technologically innovative visualization concept. Under the conditions evaluated in this study, however, equivalence to a standard microscope could not be demonstrated for routine otologic and lateral skull base surgery. The system generation investigated did not fulfill essential optical and handling requirements necessary for efficient microsurgical work, with limitations in image quality, depth perception, and system handling affecting the surgical workflow. In contrast, comparable performance was observed in selected domains such as ergonomics. While these advantages are relevant, they did not compensate for the technical limitations identified in the present study. It should be emphasized that the present findings apply to the specific system generation evaluated and the study conditions employed. Future technological developments, including newer exoscope generations and robotic holding systems, may further address current limitations. However, their clinical relevance and potential equivalence to microscopes should be evaluated in dedicated comparative studies.

## Supplementary Information

Below is the link to the electronic supplementary material.

Supplementary Material 1 (PDF 511 KB)

## Data Availability

In this study, all relevant processed data are within the paper and its supporting figures, tables, and annexes. Raw data is available on request.

## Declarations

### Permission to reproduce material from other sources

All materials and content reproduced from other sources have been appropriately credited. Permissions have been obtained for all copyrighted materials used in this manuscript and are documented in the supplementary materials.

### Use of AI tools

AI-based writing assistants (GPT 5, Open AI) were used for language polishing, style refinement, formatting, consistency checks, and support in organizing the literature. The AI provided wording suggestions and brief summaries only; it did not generate research questions, study design, data, analyses, results, or conclusions. All AI-suggested content was reviewed, corrected where necessary, and ultimately approved by the authors, who take full responsibility for the manuscript. No sensitive or personally identifiable data were entered into AI services. Use of AI complied with the journal’s policies and recommendations; the AI is not listed as an author
