# Supplementary material for: Prospective evaluation of the VITOM 3D exoscope in ear surgery compared with surgical microscopes: part II—optical performance, handling, workload and ergonomics
Source: Eur Arch Otorhinolaryngol. 2026 Mar 9;283(5):3037–50. doi: 10.1007/s00405-026-10045-x (PMC13152871; doi:10.1007/s00405-026-10045-x)
Supplement: Supplementary file 1 — Supplementary Material 1 (PDF 511 KB) [file 405_2026_10045_MOESM1_ESM.pdf]

Questionnaire for surgeons – Stapesplasty

Patient-ID:
Surgeon-ID:
Date:
Use of: O VITOM O Surgical microscope (model: ______________________)

	1	2	3	4	5	6		not applicable
**1. How satisfied were you with the overall handling of the system?**								
satisfied	O	O	O	O	O	O	unsatisfied	O
**2. How good was the overview?**								
very good	O	O	O	O	O	O	poor	O
**3. How good was the detectability of details?**								
very good	O	O	O	O	O	O	poor	O
**4. How good was the visual representation?**								
very good	O	O	O	O	O	O	poor	O
**5. How satisfied were you with the operation of the system?**								
satisfied	O	O	O	O	O	O	unsatisfied	O
**6. How satisfied were you with your posture during the surgery?**								
satisfied	O	O	O	O	O	O	unsatisfied	O
**7. How often were you obstructed by parts of the optical system?**								
all the time	O	O	O	O	O	O	never	O
**8. How satisfied were you with the depth representation of the 3D image?**								
satisfied	O	O	O	O	O	O	unsatisfied	O
**9. How bothersome did you find the 3D glasses?**								
not disturbing	O	O	O	O	O	O	very disturbing	O
**10. How bothersome did you find the optical interferences while operating via the monitor image? (Lack of ocular effect)**								
not disturbing	O	O	O	O	O	O	very disturbing	O
**11. How satisfied were you with the illumination of the field of view?**								
satisfied	O	O	O	O	O	O	unsatisfied	O

	1	2	3	4	5	6		not applicable
**12. How satisfied were you with the representation of the...:**								
**a. Chorda tympani**								
satisfied	O	O	O	O	O	O	unsatisfied	O
**b. Facial nerve canal**								
satisfied	O	O	O	O	O	O	unsatisfied	O
**c. Stapes superstructure**								
satisfied	O	O	O	O	O	O	unsatisfied	O
**d. Stapedius tendon**								
satisfied	O	O	O	O	O	O	unsatisfied	O
**e. Footplate region**								
satisfied	O	O	O	O	O	O	unsatisfied	O
**f. Stapedotomy**								
satisfied	O	O	O	O	O	O	unsatisfied	O
**g. Prosthesis insertion**								
satisfied	O	O	O	O	O	O	unsatisfied	O
**h. Prosthesis coupling**								
satisfied	O	O	O	O	O	O	unsatisfied	O

Questionnaire for surgeons – Reconstructive ear surgery

Patient-ID:
Surgeon-ID:
Date:
Use of: O VITOM O Surgical microscope (model: ______________________)

	1	2	3	4	5	6		not applicable
**1. How satisfied were you with the overall handling of the system?**								
satisfied	O	O	O	O	O	O	unsatisfied	O
**2. How good was the overview?**								
very good	O	O	O	O	O	O	poor	O
**3. How good was the detectability of details?**								
very good	O	O	O	O	O	O	poor	O
**4. How good was the visual representation?**								
very good	O	O	O	O	O	O	poor	O
**5. How satisfied were you with the operation of the system?**								
satisfied	O	O	O	O	O	O	unsatisfied	O
**6. How satisfied were you with your posture during the surgery?**								
satisfied	O	O	O	O	O	O	unsatisfied	O
**7. How often were you obstructed by parts of the optical system?**								
all the time	O	O	O	O	O	O	never	O
**8. How satisfied were you with the depth representation of the 3D image?**								
satisfied	O	O	O	O	O	O	unsatisfied	O
**9. How bothersome did you find the 3D glasses?**								
not disturbing	O	O	O	O	O	O	very disturbing	O
**10. How bothersome did you find the optical interferences while operating via the monitor image? (Lack of ocular effect)**								
not disturbing	O	O	O	O	O	O	very disturbing	O
**11. How satisfied were you with the illumination of the field of view?**								
satisfied	O	O	O	O	O	O	unsatisfied	O

	1	2	3	4	5	6		not applicable
**12. How satisfied were you with the...:**								
a. Assessment / representation of the cholesteatoma								
satisfied	O	O	O	O	O	O	unsatisfied	O
b. Assessment of the perimatrix								
satisfied	O	O	O	O	O	O	unsatisfied	O
c. Depiction of the demarcation of the cholesteatoma from the bone								
satisfied	O	O	O	O	O	O	unsatisfied	O
d. Depiction of the demarcation of the cholesteatoma from the mucosa								
satisfied	O	O	O	O	O	O	unsatisfied	O
e. Depiction of the ossicles								
satisfied	O	O	O	O	O	O	unsatisfied	O
f. Depiction of the facial nerve								
satisfied	O	O	O	O	O	O	unsatisfied	O
i. Depiction of the chorda tympani								
satisfied	O	O	O	O	O	O	unsatisfied	O
j. Depiction of prosthesis insertion								
satisfied	O	O	O	O	O	O	unsatisfied	O
k. Assessment of prosthesis coupling								
satisfied	O	O	O	O	O	O	unsatisfied	O
l. Prosthesis handling								
satisfied	O	O	O	O	O	O	unsatisfied	O

Questionnaire for surgeons – CI surgery

Patient-ID:
Surgeon-ID:
Date:
Use of: O VITOM O Surgical microscope (model: ______________________)

	1	2	3	4	5	6		not applicable
1. How satisfied were you with the overall handling of the system?								
satisfied	O	O	O	O	O	O	unsatisfied	O
2. How good was the overview?								
very good	O	O	O	O	O	O	poor	O
3. How good was the detectability of details?								
very good	O	O	O	O	O	O	poor	O
4. How good was the visual representation?								
very good	O	O	O	O	O	O	poor	O
5. How satisfied were you with the operation of the system?								
satisfied	O	O	O	O	O	O	unsatisfied	O
6. How satisfied were you with your posture during the surgery?								
satisfied	O	O	O	O	O	O	unsatisfied	O
7. How often were you obstructed by parts of the optical system?								
all the time	O	O	O	O	O	O	never	O
8. How satisfied were you with the depth representation of the 3D image?								
satisfied	O	O	O	O	O	O	unsatisfied	O
9. How bothersome did you find the 3D glasses?								
not disturbing	O	O	O	O	O	O	very disturbing	O
10. How bothersome did you find the optical interferences while operating via the monitor image? (Lack of ocular effect)								
not disturbing	O	O	O	O	O	O	very disturbing	O
11. How satisfied were you with the illumination of the field of view?								
satisfied	O	O	O	O	O	O	unsatisfied	O

	1	2	3	4	5	6		not applicable
12. How satisfied were you with the depiction of the…:								
a. Dura								
satisfied	O	O	O	O	O	O	unsatisfied	O
b. Sinus								
satisfied	O	O	O	O	O	O	unsatisfied	O
c. Posterior ear canal wall								
satisfied	O	O	O	O	O	O	unsatisfied	O
d. Lateral semicircular canal								
satisfied	O	O	O	O	O	O	unsatisfied	O
e. Malleus (in terms of anatomy)								
satisfied	O	O	O	O	O	O	unsatisfied	O
f. Facial nerve								
satisfied	O	O	O	O	O	O	unsatisfied	O
m. Chorda tympani								
satisfied	O	O	O	O	O	O	unsatisfied	O
n. Posterior tympanotomy (stapes superstructure, stapedius tendon, round window)								
satisfied	O	O	O	O	O	O	unsatisfied	O
o. Visualization of electrode insertion								
satisfied	O	O	O	O	O	O	unsatisfied	O
p. Visualization of the stapedius reflex								
satisfied	O	O	O	O	O	O	unsatisfied	O

Appendix A4 Questionnaire for the surgeon – General evaluation

Which system do you consider better in terms of overall handling?

SM better both equal VITOM better

Which system do you consider better in terms of visual representation?

SM better both equal VITOM better

Which system do you consider better in terms of ergonomics?

SM better both equal VITOM better

Which system do you consider better in terms of operation?

SM better both equal VITOM better

Appendix A5 NASA TLX Questionnaire

Mental Demand — How mentally demanding was the task?

Very Low — Very High

Physical Demand — How physically demanding was the task?

Very Low — Very High

Temporal Demand — How hurried or rushed was the pace of the task?

Very Low — Very High

Performance — How successful were you in accomplishing what you were asked to do?

Perfect — Failure

Effort — How hard did you have to work to accomplish your level of performance?

Very Low — Very High

Frustration — How insecure, discouraged, irritated, stressed, and annoyed wereyou?

Very Low — Very High

Adapted from

Hart, S. G., & Staveland, L. E. (1988). Development of NASA-TLX (Task Load Index): Results of empirical and theoretical research. In *Advances in psychology* (Vol. 52, pp. 139-183). North-Holland.

Appendix A6 LMD Questionnaire

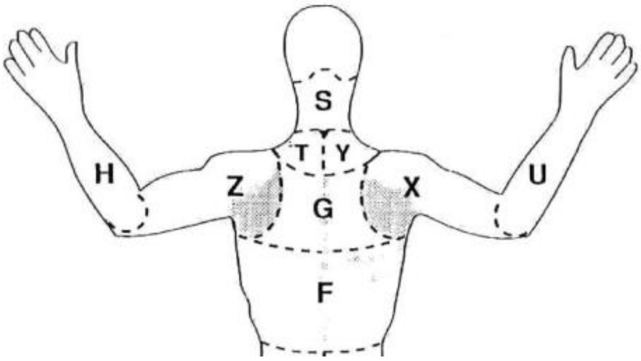

.		maximum
10	=	extreme discomfort
		(almost maximum)
9	=	
8	=	
7	=	very high discomfort
6	=	
5	=	high discomfort
4	=	somewhat high discomfort
3	=	moderate discomfort
2	=	little discomfort
1	=	very little discomfort
½	=	extremely little discomfort
0	=	no discomfort at all

Adapted from

Hamberg-van Reenen, H. H., Van Der Beek, A. J., Blatter, B. M., Van Der Grinten, M. P., Van Mechelen, W., & Bongers, P. M. (2008). Does musculoskeletal discomfort at work predict future musculoskeletal pain?. *Ergonomics*, *51*(5), 637-648.

Appendix A7 Cluster Analysis: Method, Calculation, Visualization, Python Code

To explore structural relationships between the three surgical subgroups (CI, COMwC, COMsC) across the domains General questionnaire parameters, NASA-TLX and Localized Musculoskeletal Discomfort (LMD), a hierarchical cluster analysis was performed using Ward's minimum variance method.

All calculations were implemented in Python (v3.10) using NumPy, SciPy, pandas, matplotlib, and seaborn libraries.

1. Distance Metric

The similarity between two subgroups A and B was quantified using the **Euclidean distance** between their mean item vectors:

$$d(A,B) = \sqrt{\sum_{i=1}^{n}(x_{iA} - x_{iB})^2}$$

where x_{iA} and x_{iB} denote the mean values of item i in subgroup A and B, respectively, and n is the number of items within the given domain.

2. Ward's Linkage and Increment in Sum of Squared Errors (ΔSSE)

Cluster agglomeration followed **Ward's method**, which iteratively merges clusters that result in the **minimum increase of total within-cluster variance (sum of squared errors, SSE)**.
For two clusters A and B containing n_A and n_B observations with centroids μ_A and μ_B , the increase in SSE after fusion is given by:

$$\Delta\mathrm{SSE}(A,B) = \frac{n_A n_B}{n_A + n_B} \left\|\mu_A - \mu_B\right\|^2$$

This expression represents the *Ward linkage distance* and was used as the hierarchical distance metric in the dendrogram structure.
In the present analysis, only three subgroups were included, resulting in **two hierarchical merges**:

- **Merge 1 (ΔSSE_1):** fusion of the two most similar subgroups
- **Merge 2 (ΔSSE_2):** addition of the remaining, most distinct subgroup to the cluster formed in step 1

The **ratio** of these linkage distances

$$R = \frac{\Delta\mathrm{SSE}_2}{\Delta\mathrm{SSE}_1}$$

quantifies the relative distinctness of the third subgroup. Ratios $R>1.5$ indicate a clearly isolated subgroup, whereas $R\approx1.0$ denotes comparable similarity among all three groups.

---

3. Quantitative Visualization

For each domain and visualization system, the calculated ΔSSE_1 and ΔSSE_2 values were visualized in three complementary plots:

1. **Cluster Distance Scatter Plot:**
   Depicts each domain as a point in the ΔSSE_1–ΔSSE_2 space.
   Points above the diagonal (y=x) indicate stronger separation of the third subgroup ($\Delta SSE_2 > \Delta SSE_1$).
2. **Cluster Separation Ratio Plot:**
   Summarizes the relative separation strength R across all domains and systems, where higher bars indicate greater distinctness of one subgroup.

Domain	System	Merge 1 Clusters	Merge 2 Clusters	ΔSSE 1	ΔSSE 2	Ratio (M2/M1)	Mean (CI)	Mean (COMwC)	Mean (COMsC)	Centroid (first merge)	Δmean (distinct − centroid)	Most distinct subgroup	Direction / Interpretation
General	Surgical Microscope	CI + COMsC	(CI + COMsC) + COMwC	0,271	0,771	2,84	2,12	1,84	2,09	2,11	-0,27	**COMwC**	**Lower → better rating**
General	VITOM 3D	COMwC + COMsC	(COMwC + COMsC) + CI	0,284	4,265	15,02	3,52	2,48	2,83	2,66	0,86	**CI**	**Higher → worse rating**
LMD	Surgical Microscope	COMwC + COMsC	(COMwC + COMsC) + CI	0,543	4,516	8,32	3,42	3,19	3,27	3,23	0,19	**CI**	**Higher → more strain**
LMD	VITOM 3D	CI + COMwC	(CI + COMwC) + COMsC	0,965	2,101	2,18	3,84	3,69	3	3,76	-0,76	**COMsC**	**Lower → less strain**
NASA-TLX	Surgical Microscope	CI + COMsC	(CI + COMsC) + COMwC	0,74	6,338	8,57	4,71	3,97	4,36	4,54	-0,56	**COMwC**	**Lower → lower workload**
NASA-TLX	VITOM 3D	COMwC + COMsC	(COMwC + COMsC) + CI	1,097	3,129	2,85	5,17	4,4	4,23	4,32	0,85	**CI**	**Higher → higher workload**

Table A1 Summary of cluster analysis calculations.

Python code for basic cluster analysis calculation and visualization

```
# ============================================
# 🔬 CLUSTERING + VISUALIZATION (REAL DATA)
# ============================================
import pandas as pd
import numpy as np
import matplotlib.pyplot as plt
import os


# --------------------------------------------------
# OUTPUT DIRECTORY
# --------------------------------------------------
output_dir = r"C:\Users\chris\Documents\PythonProjects\exports"
os.makedirs(output_dir, exist_ok=True)


# --------------------------------------------------
# 🔷 1) LMD data (Localized Musculoskeletal Discomfort)
# --------------------------------------------------
cols = ["CI", "COMwC", "COMsC"]


lmd_sm = pd.DataFrame({
    "CI":     [3.33, 4.22, 4.11, 2.22, 2.11, 1.25, 1.22, 3.33, 2.78],
    "COMwC":  [3.27, 3.70, 4.00, 3.22, 3.80, 1.83, 2.39, 2.78, 2.56],
    "COMsC":  [3.40, 3.30, 3.70, 3.33, 3.90, 2.10, 2.50, 3.50, 3.00]
}, index=[
    "Upper neck", "Left lower neck", "Right lower neck", "Left shoulder",
    "Right shoulder", "Left arm", "Right arm", "Upper back", "Lower back"
])


lmd_vitom = pd.DataFrame({
    "CI":     [3.44, 4.22, 4.00, 2.78, 2.85, 1.86, 1.94, 4.45, 3.80],
    "COMwC":  [3.55, 3.18, 3.55, 2.80, 3.45, 2.17, 2.30, 4.36, 4.00],
    "COMsC":  [2.65, 3.30, 3.40, 2.60, 2.90, 1.90, 2.00, 3.40, 2.90]
}, index=lmd_sm.index)
```

```
# --------------------------------------------------

# 🔷 2) NASA-TLX data

# --------------------------------------------------

nasa_sm = pd.DataFrame({

    "CI":     [5.10, 5.80, 5.22, 2.56, 5.90, 4.13],

    "COMwC":  [4.40, 5.82, 3.45, 2.29, 4.18, 1.78],

    "COMsC":  [5.11, 5.00, 4.60, 2.44, 5.50, 3.60]

}, index=["Mental demand","Physical demand","Temporal demand","Performance","Effort","Frustration"])


nasa_vitom = pd.DataFrame({

    "CI":     [5.70, 5.40, 5.20, 3.78, 5.40, 5.50],

    "COMwC":  [5.20, 5.00, 4.82, 3.13, 4.55, 3.71],

    "COMsC":  [4.50, 4.50, 4.10, 2.80, 5.00, 4.50]

}, index=nasa_sm.index)


# --------------------------------------------------

# 🔷 3) General questionnaire data

# --------------------------------------------------

gen_sm = pd.DataFrame({

    "CI":     [2.50, 2.20, 1.80, 2.00, 2.70, 3.33, 4.00, 2.00, 2.00],

    "COMwC":  [2.09, 2.18, 1.36, 1.73, 2.27, 3.73, 3.91, 1.20, 2.00],

    "COMsC":  [2.10, 1.90, 1.80, 1.80, 2.30, 3.10, 4.10, 1.86, 1.90]

}, index=[

    "Handling","Overview","Details","Optical display","Operability",

    "Body posture","Obstruction","Depth perception","Illumination"

])


gen_vitom = pd.DataFrame({

    "CI":     [3.70, 3.70, 3.70, 3.60, 3.30, 3.20, 3.70, 4.50, 4.00],

    "COMwC":  [2.55, 2.73, 3.00, 2.82, 2.55, 3.00, 4.45, 3.73, 2.91],

    "COMsC":  [2.80, 2.60, 2.70, 3.00, 2.60, 2.80, 4.00, 3.40, 2.80]

}, index=gen_sm.index)


# --------------------------------------------------

# 🔷 CLUSTERING HELPERS (Ward's method)

# --------------------------------------------------
```

```
def euclid(a, b):
    return np.linalg.norm(a - b)


def delta_sse(nA, nB, muA, muB):
    """Ward linkage increase in SSE when merging clusters A and B."""
    return (nA * nB) / (nA + nB) * (euclid(muA, muB) ** 2)


def infer_clusters(df):
    """
    Hierarchical clustering with 3 subgroups → 2 merges.
    Returns: merge labels, ΔSSE1, ΔSSE2, ratio, and the most distinct subgroup.
    """
    X = df.T.values
    labels = list(df.columns)
    clusters = {0: X[0:1,:], 1: X[1:2,:], 2: X[2:3,:]}
    sizes   = {0:1, 1:1, 2:1}
    cent    = {k: v.mean(axis=0) for k, v in clusters.items()}

    # Merge 1: smallest ΔSSE among all pairs
    pairs = [(0,1),(0,2),(1,2)]
    best, best_delta = None, np.inf
    for a,b in pairs:
        dlt = delta_sse(sizes[a], sizes[b], cent[a], cent[b])
        if dlt < best_delta:
            best_delta, best = dlt, (a,b)
    a,b = best
    merge1_delta = best_delta
    merge1_names = f"{labels[a]} + {labels[b]}"

    # Merge 2: remaining subgroup joins the merged pair
    new_key = 3
    clusters[new_key] = np.vstack([clusters[a], clusters[b]])
    sizes[new_key]   = sizes[a] + sizes[b]
    cent[new_key]    = clusters[new_key].mean(axis=0)
    del clusters[a], clusters[b], sizes[a], sizes[b], cent[a], cent[b]
    other = [k for k in [0,1,2] if k not in (a,b)][0]
    merge2_delta = delta_sse(sizes[new_key], 1, cent[new_key], X[other,:])
```

```
    merge2_names = f"({labels[a]} + {labels[b]}) + {labels[other]}"

    ratio = merge2_delta / merge1_delta if merge1_delta != 0 else np.nan
    most_distinct = labels[other]
    return merge1_names, merge2_names, merge1_delta, merge2_delta, ratio, most_distinct

# --------------------------------------------------
# 🔷 RUN CLUSTERING
# --------------------------------------------------
data_dict = {
    "LMD_SM": lmd_sm, "LMD_VITOM": lmd_vitom,
    "NASA_SM": nasa_sm, "NASA_VITOM": nasa_vitom,
    "GEN_SM": gen_sm, "GEN_VITOM": gen_vitom,
}

summary_rows = []
for key, df in data_dict.items():
    m1, m2, d1, d2, ratio, distinct = infer_clusters(df)
    if   "LMD"  in key: domain = "LMD"
    elif "NASA" in key: domain = "NASA-TLX"
    elif "GEN"  in key: domain = "General"
    else:               domain = "Other"
    system = "Surgical Microscope" if "SM" in key else "VITOM 3D"
    summary_rows.append({
        "Domain": domain,
        "System": system,
        "Merge 1 Clusters": m1,
        "Merge 2 Clusters": m2,
        "ΔSSE 1": round(d1, 3),
        "ΔSSE 2": round(d2, 3),
        "Ratio (M2/M1)": round(ratio, 2),
        "Most distinct Subgroup": distinct
    })

summary_df = pd.DataFrame(summary_rows).sort_values(by=["Domain","System"]).reset_index(drop=True)

# Save summary as German-formatted CSV (Appendix-ready for Excel in DE locale)
```

```
summary_path = os.path.join(output_dir, "Cluster_Summary_Final_DE.csv")

summary_df.to_csv(summary_path, index=False, sep=';', decimal=',', encoding='utf-8-sig')

display(summary_df)

print(f"\n✅ Summary table saved to: {summary_path}")


# --------------------------------------------------

# 🔷 VISUALIZATION (Bar, Scatter, Ratio)

# --------------------------------------------------

df = summary_df.copy()

df["System_short"] = df["System"].map({"Surgical Microscope": "SM", "VITOM 3D": "VITOM"})

df = df.sort_values(by=["Domain","System_short"]).reset_index(drop=True)

color_map = {"SM": "#1f77b4", "VITOM": "#ff7f0e"}


# Scatter Plot: ΔSSE1 (x) vs ΔSSE2 (y)

fig, ax = plt.subplots(figsize=(6,5))

for _, row in df.iterrows():

    ax.scatter(row["ΔSSE 1"], row["ΔSSE 2"], s=100,

               color=color_map[row["System_short"]],

               label=row["System"] if row["System"] not in ax.get_legend_handles_labels()[1] else "")

    ax.text(row["ΔSSE 1"]*1.02, row["ΔSSE 2"], row["Domain"], fontsize=9, va="center")

ax.plot([0, df["ΔSSE 2"].max()*1.1], [0, df["ΔSSE 2"].max()*1.1],

        "--", color="gray", lw=0.8)

ax.set_xlabel("ΔSSE₁ (Merge 1)")

ax.set_ylabel("ΔSSE₂ (Merge 2)")

ax.set_title("Cluster Variance Increments per Domain (Ward’s method)")

ax.legend()

plt.tight_layout()

plt.savefig(os.path.join(output_dir, "Cluster_Distances_Scatter.png"), dpi=300)

plt.show()


# Ratio Plot: ΔSSE2 / ΔSSE1

fig, ax = plt.subplots(figsize=(8,4))

ax.bar
```
